# Current Situation of Proton Therapy for Hodgkin Lymphoma: From Expectations to Evidence

**DOI:** 10.3390/cancers13153746

**Published:** 2021-07-26

**Authors:** Pierre Loap, Alfredo Mirandola, Ludovic De Marzi, Remi Dendale, Alberto Iannalfi, Viviana Vitolo, Amelia Barcellini, Andrea Riccardo Filippi, Barbara Alicja Jereczek-Fossa, Youlia Kirova, Ester Orlandi

**Affiliations:** 1 Department of Radiation Oncology, Institut Curie, 75005 Paris, France; ludovic.demarzi@curie.fr (L.D.M.); remi.dendale@curie.fr (R.D.); Youlia.kirova@curie.fr (Y.K.); 2 Radiation Oncology Clinical Department, National Center for Oncological Hadrontherapy (CNAO), 27100 Pavia, Italy; Alfredo.Mirandola@cnao.it (A.M.); alberto.iannalfi@cnao.it (A.I.); viviana.vitolo@cnao.it (V.V.); amelia.barcellini@cnao.it (A.B.); ester.orlandi@cnao.it (E.O.); 3 Institut Curie, PSL Research University, University Paris Saclay, INSERM LITO, 91400 Orsay, France; 4 Radiation Oncology Department, Fondazione IRCCS Policlinico San Matteo and University of Pavia, 27100 Pavia, Italy; a.filippi@smatteo.pv.it; 5 Department of Oncology and Hemato-Oncology, University of Milan, 20122 Milan, Italy; barbara.jereczek@ieo.it; 6 Division of Radiotherapy, IEO European Institute of Oncology IRCCS, 20141 Milan, Italy

**Keywords:** hodgkin lymphoma, proton therapy, NTCP model, toxicity

## Abstract

**Simple Summary:**

Hodgkin lymphoma (HL) is a highly curable disease; in this context, the limitation of late adverse events is of prime importance for the patient. Proton therapy for mediastinal HL irradiation theoretically limits secondary cancer excess risk and should reduce late toxicities compared with classical radiation therapy techniques. However, due to the limited clinical experience, strong evidence is still lacking to support proton therapy in HL management despite excellent tolerance. In addition, randomized controlled trials are probably unrealistic in this context. National and international registries may be useful to strengthen support for HL proton therapy.

**Abstract:**

Consolidative radiation therapy (RT) is of prime importance for early-stage Hodgkin lymphoma (HL) management since it significantly increases progression-free survival (PFS). Nevertheless, first-generation techniques, relying on large irradiation fields, delivered significant radiation doses to critical organs-at-risk (OARs, such as the heart, to the lung or the breasts) when treating mediastinal HL; consequently, secondary cancers, and cardiac and lung toxicity were substantially increased. Fortunately, HL RT has drastically evolved and, nowadays, state-of-the-art RT techniques efficiently spare critical organs-at-risks without altering local control or overall survival. Recently, proton therapy has been evaluated for mediastinal HL treatment, due to its possibility to significantly reduce integral dose to OARs, which is expected to limit second neoplasm risk and reduce late toxicity. Nevertheless, clinical experience for this recent technique is still limited worldwide. Based on current literature, this critical review aims to examine the current practice of proton therapy for mediastinal HL irradiation.

## 1. Introduction

Hodgkin lymphoma (HL) is a rare hematologic malignancy with an estimated incidence of 2.7–2.8 cases per 100,000 person-year [1] and is characterized by a high curative rate, evaluated between 80% and 90% [2]. For favorable and unfavorable early-stage HL, consolidative radiation therapy (RT) is currently considered as a gold standard since it significantly improves progression-free survival [3]. More controversially, RT can also be considered for some advanced-stage HL, as well as in the context of relapsed or refractory HL. Unfortunately, first generation radiation techniques that heavily relied on two-dimensional planning and large extended fields were associated with a significantly increased morbidity for mediastinal HL irradiation, notably including secondary cancers, and cardiac and pulmonary toxicity [4]. Treatment planning evolutions were subsequently achieved and, nowadays, widespread use of smaller target volumes limited to the initially involved sites or to the involved nodes allowed a substantial reduction of integral dose delivered to the patient [5]. Additionally, drastic technical progress was made to spare organs-at-risks (OAR) without altering local tumor control or patient survival. Such evolutions included generalization of intensity-modulated radiation therapy (IMRT) and democratization of respiratory control such as gating or deep-inspiration breath-hold (DIBH) [6]. The issue around radiation-related toxicity after HL treatment has significantly evolved over the years. Proton therapy has recently been proposed to further reduce late toxicity in the context of mediastinal HL irradiation. Proton beams deliver most of their energy at the end of their ranges, translating into a limited distant-to-target dose deposition compared with IMRT. For mediastinal HL, proton therapy has thus demonstrated a major dosimetric benefit for cardiac sparing [7] while decreasing the integral dose to the lungs and to the breasts. In addition, newer IMRT techniques for HL management, such as volumetric modulated arctherapy (VMAT) or helical tomotherapy sensibly increase low-dose exposure to OARs which had been debatably suspected to potentially increase secondary malignancies, based on low-dose radiation data from A bomb survivors [8]; however, other biological models failed to demonstrate any risk increase of second cancers for low-dose exposure [9] and this issue is still controversial. Consequently, proton therapy might be an excellent therapeutic option for selected mediastinal HL patients who would benefit from reduced radiation exposure to the OARs; such patients could correspond to those with significant baseline cardiac risk factors or young female patients. However, while expectations around HL proton therapy for late toxicity reduction are high, clinical experience is still limited. The purpose of this review is to provide a contextualized analysis of the expectations and of the current clinical evidence for mediastinal HL management with proton therapy.

## 2. Modeling the Benefit of Proton Therapy for Hodgkin Lymphoma Irradiation

**Figure 1:**
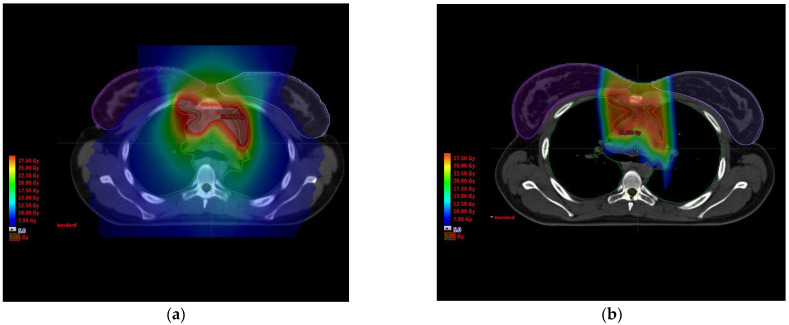
Example of organ-at-risk (OAR) sparing with proton therapy for Hodgkin lymphoma. This 24-year-old patient had to be treated to the dose of 30.6 Gy to the PTV (red), partly localized in the inferior anterior mediastinum, under the origin of the left main coronary artery. A comparative dosimetry was realized between DIBH-VMAT plan (**a**) and proton therapy (**b**). Proton therapy reduced radiation exposure to the breasts (delineated in purple), to the lungs (delineated in green), to the heart and to the esophagus, compared with VMAT; this may translate into fewer OAR late toxicities (and potentially fewer secondary cancers). In particular, the mean heart dose was reduced from 3.7 Gy with VMAT to 2.6 Gy with proton therapy, which might reduce late radiation-induced cardiotoxicity. This patient was finally treated using DIBH involved site proton therapy.

Due to their physical properties, proton beams release most of the energy towards the end on their range, a phenomenon known as the “Bragg peak”, which allows to substantially reduce distant-to-target dose deposition compared with classic photon radiation therapy techniques. Consequently, the theoretical benefit of proton therapy for HL irradiation is based on the substantial OAR sparing compared with modern photon radiation therapy techniques. Figure 1 provides a typical example of significant OAR sparing with proton therapy compared with VMAT. The plan comparison was done with optimized multi-arc VMAT technique with the same dose constraints.

### 2.1. Reduction of Secondary Neoplasms 

Hodgkin lymphoma (HL) survivors have a significantly increased risk of secondary neoplasms, which may result from both chemotherapy regimens and radiotherapy. Based on a cohort of 3905 HL patients with a median follow-up of 19.1 years, Schaapveld et al. [10] estimated that the standardized incidence ratio (SIR) of secondary cancers was 4.6. The risk was particularly increased for non-Hodgkin lymphomas (SIR = 13.4), thyroid cancers (SIR = 14.0) and mesothelioma (SIR = 15.1); however, the greatest epidemiological burden corresponded to breast cancers, with an excess absolute risk (EAR) of 54.3 cases for 10,000 person-years, and to lung cancers with an EAR of 24.6 cases for 10,000 person-years. The benefit of HL proton therapy for secondary neoplasm risk reduction was expected based on the principle that lower doses were associated with a reduced risk of radiation-induced cancer [11]. This concept of a linear no-threshold model has been proposed by retrospective analyses of second cancer incidence in A-bomb survivors in Japan; on this basis, mathematical models predicting radiation-induced secondary cancers were proposed by Schneider et al. (a mechanistic model using the linear quadratic formula) [12] or Sachs & Brenner (a biological-based initiation-inactivation-proliferation model) [13].

Based on the Schneider model and taking as a reference the anterior-posterior three-dimensional (3D) RT (3D-RT) planning, Cella et al. [14] found that HL proton therapy was associated with a significantly reduced relative risk (RR) of secondary breast cancers (RR = 0.3–0.7) compared with 3D-RT; while a benefit for secondary lung cancers was also observed (RR = 0.6–0.7), this was not the case for thyroid cancers. Taking the “butterfly” IMRT technique [15] as a reference, König et al. [16] similarly demonstrated that secondary breast, lung, and esophageal cancers could be reduced by 56.4%, 54.4% and 24.4% respectively. Scorsetti et al. [17] evidenced that proton therapy could also reduce thyroid cancers compared with VMAT; however, this benefit is expected to be limited to an EAR reduction of 0.2 for 10,000 person-years. However, modern RT techniques currently used for HL irradiation (“butterfly” IMRT, VMAT or tomotherapy) may be associated with an increased low-dose bath compared with 3D-RT [8], which usually results from a compromise between heart and breast sparing, but which might be associated with uncertainties regarding secondary malignancy risk. Timlin et al. [18] estimated that the overall cumulated risk of secondary cancers after HL RT (including breast, esophagus, heart, liver, lung, pharyngeal, spinal cord, stomach, thyroid, bone, and soft tissues malignancies) would be significantly lowered with proton therapy compared with IMRT or VMAT (reduction of EAR of 40% and 28% respectively) but would be somewhat similar compared with 3D-RT (EAR reduction of 4%). However, it should be stressed that when applying strict dose constraints to the breast [19], risk of secondary breast cancer is similar between 3D-RT and VMAT [20]. Consequently, a comparison between 3D-RT, optimized multi-arc VMAT and proton therapy using strictly similar dose constraints to the breasts might be considered in the future to precise these uncertainties.

### 2.2. Reduction of Radiation-Induced Cardiotoxicity

Cardiotoxicity is another major concern for HL survivors. Van Nilwegen et al. [21] estimated on a cohort of 2524 patients with a median follow-up of 20 years that these patients had a 4–6-fold increase of heart failure (HF) or congestive heart failure (CHF). The cumulative incidences of CHD, HF, and valvular heart disease (VHD) were 20%, 11% and 31% respectively. Mediastinal RT was found to increase CHD with a hazard ratio (HR) of 2.7, VHD with a HR of 6.6 and HF with a RR of 2.7. Significant interactions were found with anthracyclines-based chemotherapy use and smoking status. 

Long-term follow-up of patients treated with RT for diverse types of cancer allowed the development of normal tissue complication probability (NTCP) models to estimate the risk of radiation-induced side effects. In particular, these models could be used to estimate radiation-induced cardiotoxicity risk: Gagliardi et al. summarized available cardiac NTCP models [22] which focused on cardiac mortality (based on a breast and an HL cohort), pericarditis (based on historical combined data and an esophagus cancer cohort) and cardiac perfusion defects (based on an HL cohort). In addition, an NTCP model for VHD risk estimation has been developed with an HL cohort, based on lung and heart dosimetric parameters [23]. However, none of these cardiac NTCP models consider cardiac substructures. Yet, Hoppe et al. [24] demonstrated that, for HL proton therapy, gross heart dosimetric parameters, such as mean heart dose (MHD), were not representative of radiation exposure to potentially critical cardiac substructures: correlation coefficients between MHD and cardiac substructures were weak for intensity-modulated proton therapy (IMPT); for instance, r = 0.5 for the tricuspid valve and r = 0.52 for the mitral valve. In this respect, current NTCP cardiotoxicity models, which are all developed with photon-based RT techniques, could be misleading when applied to proton therapy. Nevertheless, dosimetric comparison between HL proton therapy and photon techniques (3D or IMRT) demonstrated a significant gain on all cardiac substructures (cardiac cavities, valves, and coronary arteries) [7], and Taparra et al. [25] found that the superiority of IMPT over IMRT at the substructure level was conserved even with ECG-gated cardiac substructure sparing (CSS) planning. An example of comparative dosimetry between IMPT and IMRT is provided in Figure 1.

However, this apparent dosimetric superiority of proton therapy over photon techniques does not necessarily translate into clinical benefits, according to current NTCP models. Toltz et al. [26] did not find any significant reduction of cardiac mortality between free-breathing (FB) IMPT and FB-helical tomotherapy, unless the target volumes were strictly localized anteriorly to the heart, but the presence of confounding factors (such as smoking, HTA, obesity, family history or chemotherapy) in the study population could have affected the conclusions of this study. Similarly, Rechner et al. [27] failed to demonstrate a benefit on cardiac mortality when IMPT (FB or DIBH) was compared with deep-inspiration breath-hold (DIBH)-IMRT. Scorsetti et al. [17] demonstrated a statistically significant reduction of cardiac mortality, IHD and HF with DIBH-IMPT compared with DIBH-VMAT; the absolute benefit on cardiac mortality was, however, limited (0.16% for IMRT vs. 0.15% for proton therapy, *p* = 0.03).

### 2.3. Considerations on Other Organs-at-Risk

HL RT is associated with an increased risk of radiation pneumonitis. Fox et al. [28] found that mean lung dose greater than 13.5 Gy and lung V20Gy > 33.5% were predictive of radiation-induced pneumonitis for HL RT; the risk was increased in pre-transplant situations. Lyman-Kutcher-Burman (LKB) NTCP models for radiation pneumonitis were derived from a thymoma cohort by Moiseenko et al. [29] and from breast, lymphoma, and non-small cell lung cancer (NSCLC) cohorts by Seppenwoolde et al. [30]. Based on these models, Scorsetti et al. [17] estimated that DIBH-IMPT could significantly decrease radiation-induced pneumonitis (either symptomatic or radiographic) compared with DIBH-VMAT. This benefit, while statistically significant, was clinically limited (EAR reduction between 0.09% and 1.09%). In practice, with optimized multi-arc VMAT techniques, it is very uncommon to observe V20 above 30% or mean lung dose above 13.5 Gy, and, consequently, radiation pneumonitis is not seen any longer in daily practice with modern techniques. The potential benefit of proton therapy with regards to radiation-induced lung toxicity might thus be clinically inexistant in the modern era.

NTCP models were proposed for radiation-induced esophagitis. Chapet et al. [31] developed an LKB model based on a cohort of NSCLC patients treated with photon RT. Based on this model, Scorsetti et al. [17] found that proton therapy could reduce radiation-induced esophagitis probability from 2.61% with VMAT to 2.47% with proton therapy. More recently, Wang et al. [32] proposed an NTCP model based on a cohort of 328 NSCLC patients treated with proton therapy; to this date, this model has not been used in HL dosimetric studies.

Finally, an NTCP model has been developed by Palma et al. [33] for severe radiation dermatitis based on IMRT and IMPT on NSCLC cohort. Cutaneous toxicity has not been evaluated specifically for HL proton therapy, but Fellin et al. [34] found that breast proton therapy could increase skin morbidity compared with IMRT if the skin were not considered as an OAR. Delivered doses for HL lymphoma are usually lower than those for breast RT and skin toxicity for HL irradiation is usually not seen in daily practice with optimized IMRT; nevertheless, dosimetric studies could be useful to evaluate this potential issue in the context of HL irradiation with proton beams.

## 3. Current Evidence for Hodgkin Lymphoma Proton Therapy

### 3.1. Clinical Tolerance and Efficacy Data

To this date, published HL proton therapy trials are single-armed; whether the theoretical benefit of particle therapy in terms of late toxicity would be found in practice is still an open question. Available clinical data from published HL proton therapy studies are provided in Table 1. 

Hoppe et al. [35] reported the first outcomes on a phase II trial including 15 HL patients treated with involved node proton therapy in a consolidative setting. Doses ranged between 15 Gy and 39.6 Gy. With a median follow of 37 months, the 3-year relapse-free survival (RFS) and event-free survival (EFS) were respectively 93% and 87%. No grade 3–4 toxicity was observed. A subsequent report on a larger cohort of 138 patients with a median follow-up of 32 months [36] confirmed the efficacy of HL proton therapy, with a 3-year RFS of 92%, and its excellent tolerance profile, without grade 3-4 treatment-related toxicity. Similarly, no grade 3–4 pulmonary toxicity was observed by Nanda et al. [37] on 50 HL patients (in a cohort of 59 lymphoma patients); while three early pulmonary grade 2 adverse events were described in this study, there were no ≥grade 2 late toxicity. Ntentas et al. [38] did not observe any grade 3–4 toxicity on a cohort of 21 patients treated with involved site proton therapy, with a median follow-up of 24 months. Finally, the tolerance profile of proton therapy in the case of relapse/refractory (r/r) HL has also been evaluated by Tseng et al. [39] on a cohort of 85 r/r lymphoma patients (including 56 HL patients) treated to a median dose of 36 Gy. With a median follow-up of 26.3 months, no grade 3–4 pulmonary toxicity was observed; grade 2 pneumonitis (12.8% of the patients) could be predicted based on mean lung dose (MLD), lung V5Gy and V20Gy. All these trials compared favorably with IMRT, for which 4.5% of grade 3 toxicities have been reported in the consolidative setting and 12.5% for r/r HL [40]. 

**Table 1:** Published clinical data (2014–2021) on radiation exposure to organs-at-risk for Hodgkin lymphoma (HL) patients treated with proton therapy. Nanda et al. [37] and Tseng et al. [39] included non-Hodgkin lymphoma (NHL) patients in their studies. Pts.: patients. r/r: relapse refractory. RFS: relapse-free survival. EFS: event-free survival. OS: overall survival. LVEF: left ventricle ejection fraction. DLCO: diffusing capacity of lung for carbon monoxide.

Study	Size	Situation	Dose (Gy)	Follow-Up	Efficacy	Tolerance
Hoppe (2014) [35]	15 pts.	First line	15–25.5 (children) 30.6–39.6 (adults)	37 months (26–55)	3-year RFS: 93% 3-year EFS: 87%	No grade 3–4 toxicity (acute or late)
Hoppe (2017) [36]	138 pts.	First line	21 (15–36) (children). 30.6 (20–45) (adults)	32 months (5–92)	3-year RFS: 92% (96% for adults; 87% for children).	No grade 3–4 toxicity (acute or late)
Nanda (2017) [37]	50 pts. (+9 NHL pts.)	First line and r/r disease	30.6 (15–45)	24.1 months (6–82)	NA	Acute grade 2 pulmonary toxicity: cough (3 pts) ± pneumonitis (1 pt) ± dyspnea (2 pts). No grade 3–4 toxicity (acute or late)
Ntentas (2019) [38]	21 pts.	First line	30	24 months (13–38)	No recurrence. No disease progression	No grade 3–4 toxicity (acute or late)
Tseng (2021) [39]	56 pts. (+29 NHL pts.)	r/r disease	36 (20–45)	25.6 months (0.9–113.4)	2-year PFS: 73%. 2-year OS: 91%	12.8% of grade 2 pneumonitis. No grade 3–4 toxicity (acute or late)
Bates (2019) [41]	5 pts.	First line	36 (30.6–39.6)	60 months	NA	No grade 3 (acute or late). No late cardiac symptomatology. 5-year LVEF: 60% (52%–61%).
O’steen (2019) [42]	15 pts.	First line	30.6 (21–39.6)	12 months	NA	1-year DLCO: 95.7%. 1-year Forced vital capacity: 98.2%. 1-year mean forced expiratory volume (1 s): 97%.

In addition, late asymptomatic cardiac and pulmonary toxicities have been specifically scrutinized using paraclinical exams by two studies. Bates et al. [41] followed five patients with magnetic resonance imaging (MRI), echocardiography and BNP dosage. With a median follow-up of 5 years, no changes were observed on MRI; however, the use of anthracycline-based regimen was associated with a mild asymptomatic left ventricle ejection fraction (LVEF) decline. O’steen et al. [42] measured the forced vital capacity and the forced expiratory volume in one second on 15 patients at one-year follow-up and did not find any significant change. Indeed, the follow-up is still too limited to conclude to a potential benefit on late cardiac or pulmonary toxicity or a reduction of second cancer incidence, but it appears to this point that HL proton therapy is well tolerated when indirectly compared to historical photon RT data.

### 3.2. Radiation Exposure to Organs-at-Risk

Since no randomized controlled trials between proton therapy and IMRT have been conducted, dosimetric comparison studies between both techniques relied on a retrospective replanning of one of these two modalities. Such replanning is associated with an inherent risk of bias. Nevertheless, by pooling all available dosimetric HL comparison studies between proton therapy and photon RT, Tseng et al. [43] estimated that the average absolute dosimetric gain with proton therapy would be of 1.8 Gy for esophagus, 2.24 Gy for heart, 2.09 Gy for thyroid, 2.45 Gy for breast and 3.28 Gy for lungs. However, they highlighted the non-homogeneity of available studies, especially concerning the use of non-optimal photon planning and variability in volume definition. Whether this dosimetric gain “in silico” would finally translate into a clinical benefit for the patient in daily practice will be answered by long-term follow-up of HL proton therapy studies.

Available dosimetric data from published HL proton therapy studies are provided in Table 2. 

On a cohort of 15 patients treated with involved node proton therapy, Hoppe et al. [35] reported an MHD of 8.9 Gy, an MLD of 7.1 Gy, a mean dose to the breast of 4.3 Gy, a mean dose to the thyroid of 15.8 Gy and a mean dose to the esophagus of 13.4 Gy. Ntentas et al. [38] found somewhat lower doses to the OAR with an MHD of 7.7 Gy, an MLD of 5.3 Gy, a mean dose to the left ventricle (LV) of 2.0 Gy, a mean dose to the breast of 1.6 Gy and a mean dose to the esophagus of 14.2 Gy. Nanda et al. [37] described an MLD of 7.2 Gy. Tseng et al. [39] reported an MHD of 7.7 Gy and an MLD of 7.3 Gy. Bates et al. [41] reported highly variable dosimetric values, ranging between 2.1 Gy and 19.7 Gy for MHD, between 2.9 Gy and 33.4 Gy for the left anterior descending coronary artery (LADCA), and between 0.5 Gy and 27.9 Gy for the LV. 

It should be noted that the international lymphoma radiation oncology group (ILROG) mean dose recommendations [44] are less than 5 Gy for the heart, the left ventricle, and the other cardiac substructures, less than 4 Gy for the breasts and less than 10 Gy for the lungs. Consequently, while the MLD and the mean dose to the breasts observed in the available HL proton therapy studies were usually lower than the upper limits proposed by the ILROG guidelines, MHD was often higher than the recommended dose constraint. 

### 3.3. The Need for a Longer Follow-Up

The clinical data for HL proton therapy consequently lack long-term follow-up and are relatively heterogeneous from a dosimetric point of view. In addition, proton therapy techniques continue to evolve; pencil beam scanning is gradually replacing passive scattering, and robust optimization methods and Monte-Carlo calculation algorithms are being currently generalized in treatment planning [45]. Yet, the development of NTCP models need long-term follow-up and robust dosimetric data. While acute pneumonitis risk for HL proton therapy might be extrapolated from r/r HL proton therapy study by Tseng et al. [39], prediction of late toxicity risk could currently solely rely on photon NTCP models, which completely disregards specific radiobiological effect of proton beams [46] and relative biological effectiveness (RBE) variation issues [47,48]. There is consequently a need for larger population HL proton therapy studies to evaluate long-term adverse events and propose toxicity prediction models. The current registered ongoing study is the NCT03969693 trial, a prospective observational study with cardio-pulmonary monitoring which should include 50 patients. However, a larger population would probably be required to develop robust cardiac or pulmonary NTCP models.

## 4. Randomized Controlled Trials for Hodgkin Lymphoma Proton Therapy: Challenges and Pitfalls

### 4.1. Epidemiological Considerations 

Randomized controlled trials (RCTs) are currently considered the gold standard in the era of evidenced-based medicine. However, contrasting with current RCT recruiting for breast cancer proton therapy [49], the development of an RCT for HL proton therapy is challenging since mediastinal HL is a rare malignancy. Townsend et al. [1] reported an incidence of 2.7–2.8 cases per 100,000 person-year, this corresponds approximately to 1700 new cases in the United Kingdom per year (compared to 50,000 breast cancers). According to Cancer Research UK [50], the proportion of limited-stage I-II HL lymphoma represents 55% of all cases; Filly et al. [51] estimated that 67% of all HL patients had a thoracic involvement. Therefore, for a country like the United Kingdom, it could be estimated that only 600 to 700 HL patients per year have a localization amenable to proton therapy in a consolidative setting. Recruitment for a potential HL proton therapy RCT is consequently expected to be challenging.

### 4.2. Defining Relevant Clinical Endpoints

In addition, statistical hypotheses would be difficult to define a priori. On a cohort of 125 irradiated HL patients, Hahn et al. [52] observed 44 late cardiac adverse events, 70% of which were ischemic; however, treatment was delivered using outdated 2D techniques. Leeuwen et al. [53] estimated that the cardiotoxicity risk increased after 5 to 35 years of follow-up and secondary cancer incidence after 5 to 15 years, remaining elevated for at least 40 years. Nevertheless, evaluating the potential cardiotoxicity risk reduction with proton therapy, based on the improved cardiac substructure sparing capacities of this technique, might be a clinically relevant endpoint [54].

On the other hand, the toxicity follow-up for HL VMAT, helical tomotherapy or IMRT is still limited, since these techniques are recent, and insufficient in any case to have a precise estimation of late radiation-induced toxicity with them; most of the clinical experience rely on limited size series [6]. In addition, it should be stressed the stochastic effect on carcinogenesis of low-dose bath is still unknown in practice [55] and the risk of second cancers with rotational IMRT is currently subject to intense debate [56]. The optimal population size and follow-up duration for a hypothetic HL proton therapy RCT would consequently be challenging to evaluate.

### 4.3. The Ethical Aspects of RCTs for Proton Therapy 

The necessity of proton therapy RCT has been debated on an ethical point of view for a decade. Glatsein et al. [57] underlined the uncertainties about proton therapy efficacy and the importance of a measurable benefit (either enhanced anti-tumoral efficacy or toxicity reduction) to justify the financial cost of this technique; overall survival was proposed as a relevant endpoint for proton therapy RCT trials. Indeed, in a curable disease like HL, other endpoints would have to be considered, such as toxicity reduction.

On the other hand, Goitein et al. [58] considered that since proton therapy was associated with a better OAR sparing, there would be a high probability that it would be beneficial for the patient in almost all situations. There would consequently be an a priori unethical equipoise disequilibrium between the proton therapy and classic RT arms in such an RCT. This affirmation could be currently backed by a multiple tumor-site proton therapy RCT by Baumann et al. [59] which demonstrated that protons reduced acute adverse events with the same oncological outcome on a large population consisting of head and neck, pulmonary, cerebral, esophageal, gastric, rectal and pancreatic cancer patients. Bekelman et al. [60] underlined that insurance coverage inequalities (which is the case for proton therapy) may led to non-representativeness and hamper pace of enrollment of an RCT.

In any case, proton therapy is currently becoming cheaper and more accessible [61]; cost-effectiveness analyses might probably be less relevant to justify the implementation of this technique in the future. Nevertheless, to this date, identifying the best clinical indications is currently of prime importance due to the currently limited resources [56]. 

### 4.4. Does Evidenced-Based HL Proton Therapy Need Randomized Trials?

A potential HL proton therapy RCT would consequently face recruitment issues, statistical difficulties, and possible ethical debate. Barton et al. [62] argued that, when RCT would be considered unethical or unfeasible, high-quality observational studies might improve evidence level thanks to a larger size and greater representativeness of the study population. Large observational studies may be more sensitive to detect rare adverse events. Consequently, a convenient solution could be to develop national or international registries of HL patients treated with proton therapy and photon techniques to precisely evaluate outcomes and toxicities at a longer follow-up [63].

## 5. Discussion

Proton radiotherapy, taking advantage of proton beam physical properties, delivers targeted irradiation with minimal off-target dose scatter to adjacent tissues and OAR. It is consequently a promising approach for irradiation of HL patients who require RT. While RT was the first evaluated curative treatment of early-stage HL, the off-target radiation exposure resulted in potentially fatal RT-related toxicities, such as cardiovascular events or second primary malignancies that substantially increased with follow-up, particularly after 10 years [4]. 

In recent years, RT has been delivered to smaller fields, following chemotherapy, compared with total or subtotal nodal irradiation fields. Nevertheless, current practice of ISRT or INRT for localized HL usually consists of irradiation to all initially involved sites, regardless of the tumoral response to chemotherapy. Reduced radiation fields to residual sites only was evaluated in the HD15 study of the German Hodgkin Study Group for advanced HL [64]. Similarly, for early-stage HL, investigators at Memorial Sloan Kettering Cancer Center suggested that reduced fields might be sufficient for areas of remaining masses that were PET-positive following chemotherapy (residual site RT, RSRT), particularly in patients with bulky mediastinal disease: this concept was tested in newly diagnosed HL patients with bulky stage HL (IIA/BX) after four cycles of brentuximab vedotin, doxorubicin, vinblastine, and dacarbazine (BV-AVD) [65]. Most patients were PET- or biopsy-negative after chemotherapy and were randomized to four cohorts: 25 patients to 30 Gy ISRT (cohort 1), 28 patients to 20 Gy ISRT (cohort 2), 29 patients to 30 Gy RSRT (cohort 3) and 24 patients to no further treatment (cohort 4). Complete response rates were 93–100% among the four cohorts; 2-year progression-free survivals for cohorts 1 to 4 were 93%, 97%, 90% and 97%, respectively. While this study was not powered to show efficacy differences, it suggested that achievement of complete response after completion of all treatments with reduced field RT or without RT could be predictive of a good short-term outcome. In the FITIL/FIL HD 0607 trial [66], patients with newly diagnosed stages IIX, III and IV HL were treated with two cycles of ABVD. Patients who were interim PET-negative received four additional cycles of ABVD while those who were interim PET-positive received escalated BEACOPP. Interim and end-of-treatment PET-negative patients were randomized to consolidation RT or no further treatment. At a 5-year median follow-up time, no difference in PFS or OS was observed. Consequently, interim and end-of-treatment PET negativity seems to identify a significant population of newly diagnosed HL patients who may not require consolidation RT.

Reducing the size of the treatment fields and minimizing off-target dose deposition represents a logical way to reduce radiation-induced toxicity. This can be achieved with modern photon beam techniques, such as rotational VMAT or helical tomotherapy, which reduce OAR exposure compared with 3D-RT [67] and increase treatment conformation [68]. In clinical practice, Besson et al. demonstrated that adoption of IMRT for HL irradiation reduced acute toxicities compared with 3D-RT (55% versus 71.4% for all grade toxicities, respectively) [6]. While proton therapy may further reduce OAR radiation exposure, whether or not this additional dosimetric benefit is clinically important enough to justify the expense of proton therapy is still an open question. 

## 6. Conclusions

In conclusion, proton therapy for HL irradiation has the potential to reduce radiation exposure to several OARs, including cardiac substructures, which should significantly reduce late radiation-induced toxicities and secondary cancers. However, the clinical benefit of proton therapy is still uncertain and difficult to evaluate in practice, due to the limited follow-up, the presence of multiple confounding clinical variables, and the generalization of modern, highly conformal photon techniques such as DIBH-VMAT. While randomized controlled trials for HL proton therapy are probably unrealistic, the development of clinical registries might undoubtedly help to elucidate current uncertainties concerning this promising technique.

## Figures and Tables

**Table 2:** Published dosimetric data (2014–2021) on radiation exposure to organs-at-risk for Hodgkin lymphoma (HL) patients treated with proton therapy. Pts: patients. LAD: left anterior descending coronary artery. LV: left ventricle. RV: right ventricle. Nanda et al. [37] and Tseng et al. [39] included non-Hodgkin lymphoma (NHL) patients in their studies. Grey areas: dosimetric data not available.

Study	Size	Average Mean Dose (Gy)							
		Heart	LAD	LV	RV	Lung	Breast	Thyroid	Esophagus
Hoppe (2014) [35]	15 pts.	16.5				11.6	6.3	19.3	20.3
Bates (2019) [41]	5 pts.	10.6	16.4	6.5	11.7				
Ntentas (2019) [38]	21 pts.			2.0		5.3	1.6	24.1	14.2
Nanda (2017) [37]	50 pts. (+9 NHL pts.)					7.2			
O’steen (2019) [42]	15 pts.					8.0			
Tseng (2021) [39]	56 pts. (+29 NHL pts.)	7.7				7.3			
Pooled results		9.1		2.9		7.3	3.6	22.1	16.7

## Abbreviations

CT	Compute tomography
DIBH	Deep inspiration breath-hold
EAR	Excess absolute risk
FB	Free-breathing
HL	Hodgkin lymphoma
IMPT	Intensity modulated proton therapy
IMRT	Intensity modulated radiation therapy
INRT	Involved-node radiation therapy
ISRT	Involved-site radiation therapy
NTCP	Normal tissue complication probability
OAR	Organ-at-risk
RBE	Relative biological effectiveness
RR	Relative risk
RT	Radiation therapy
VMAT	Volumetric modulated arc therapy
